# Electric-Field Tunable Anisotropic *g*‑Factor Induced by Spin Pumping

**DOI:** 10.1021/acs.nanolett.5c05536

**Published:** 2026-02-20

**Authors:** Jian Shao, Matthias Kronseder, Jianping Guo, Maximilian Mangold, Dong Pan, Thomas N. G. Meier, Weiwei Zhao, Jianhua Zhao, Christian H. Back, Lin Chen

**Affiliations:** † Sauvage Laboratory for Smart Materials, School of Integrated Circuit, 529484 Harbin Institute of Technology, Shenzhen 518055, China; ‡ Department of Physics, 9184 Technical University of Munich, 85748 Garching b. Munich, Germany; § Institute of Experimental and Applied Physics, 9147 University of Regensburg, 93049 Regensburg, Germany; ∥ State Key Laboratory of Semiconductor Physics and Chip Technologies, Institute of Semiconductors, Chinese Academy of Science, Beijing 100083, China; ⊥ National Key Laboratory of Spintronics, Hangzhou International Innovation Institute, Beihang University, 11115 Hangzhou, China; # Munich Center for Quantum Science and Technology (MCQST), 80799 Munich, Germany; ∇ Center for Quantum Engineering (ZQE), 9184 Technical University of Munich, 85748 Garching b. Munich, Germany

**Keywords:** spin pumping, 2DEG, Landé g-factor, spin−orbit interaction, electric-field control of magnetism

## Abstract

Spin pumping, a well-established phenomenon where the precessing magnetization of a ferromagnet (FM) injects a pure spin current into an adjacent nonmagnetic layer, is characteristically identified by the enhancement of magnetic damping in the FM. Theoretical work has also suggested that spin pumping can affect the Landé *g*-factor—another key parameter of ferromagnetic materials besides the magnetic damping. However, experimental evidence of this effect has remained limited. Here, we demonstrate that spin pumping can lead to significant modulation of the *g*-factor in a Py/AlO_*x*_/STO two-dimensional electron gas (2DEG) system with strong spin–orbit interaction. Specifically, we observe an anisotropic *g*-factor with 2-fold symmetry below the onset temperature of the 2DEG, and furthermore this anisotropy can be tuned when applying an external electric field. This work uncovers a previously overlooked effect and offers new possibilities for controlling magnetization dynamics in systems with a reduced dimension.

## Graphical abstract

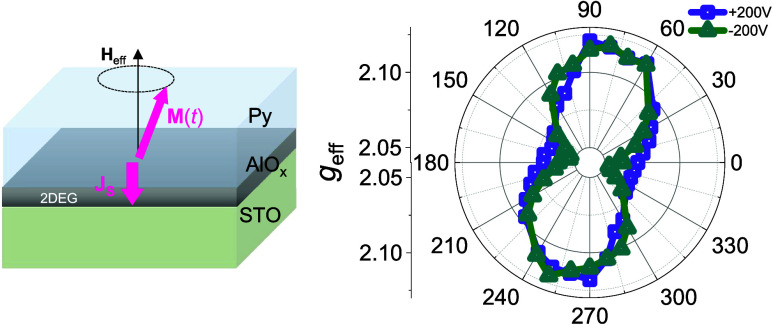

The magnetization dynamics of a ferromagnet (FM) is determined by the phenomenological Landau-Lifshitz-Gilbert equation^1^

1 $$\frac{dM}{dt}=‐\gamma M\times \mu_{0}H_{\mathrm{eff}}+\frac{\alpha }{M}M\times \frac{dM}{dt}$$

where **M** is the magnetization, *t* the time, γ (= *gμ*_B_/*ℏ*) the gyromagnetic ratio, *g* the Landé *g*-factor, μ_B_ the Bohr magneton, *ℏ* the reduced Planck constant, μ_0_ the magnetic constant, **H**_eff_ the effective magnetic-field, and α the damping constant of the FM. In FM/heavy metal (HM) bilayers, the magnetization dynamics of FM pumps spin currents that carry angular momentum and energy into the adjacent HM. The angular momentum relaxes due to the strong spin–orbit interaction (SOI) in the HM and leads to an enhancement of the damping in comparison with pure FM.^2,3^ According to the theory of spin pumping, the modified damping α_eff_ is given by^2,4^

2 αeff=geffg[α+ℏγ4πMtRe(g↑↓)]

where *t* is the thickness of FM, Re­(*g* ^↑↓^) (always positive) the real part of the complex mixing conductance *g* ^↑↓^ at the FM/HM interface, and *g*_eff_ the modified the Landé *g*-factor by spin pumping. The magnitude of *g*_eff_ is expressed as^2,4^

3 geff=g[1+ℏγ4πMtIm(g↑↓)]

where Im­(*g* ^↑↓^) is the imaginary part of *g* ^↑↓^. For ohmic FM/HM interfaces, first-principal calculations show that the sign of Im­(*g* ^↑↓^) depends on the details of the structure (e.g., thickness as well as the choice of FM) but the magnitude of |Im­(*g* ^↑↓^)| is about one order smaller than Re­(*g* ^↑↓^).^4–10^ Therefore, it is generally believed that Im­(*g* ^↑↓^) is negligibly small and there is no modulation of *g*-factor in FM/HM bilayers.

On the other hand, the reciprocal effect of spin pumping is the current-induced spin–orbit torques in FM/HM bilayers, where a dc current in HM generates additional damping-like **τ**_DL_ and field-like **τ**_FL_ torques acting on the FM.^11^ The spin transport model utilizing the drift-diffusion approximation^12,13^ shows that **τ**_DL_ is related to Re­(*g* ^↑↓^) while **τ**_FL_ is proportional to Im­(*g* ^↑↓^). The existence of **τ**_FL_ has been confirmed in a wide range of FM/HM bilayers by various experimental methods with its magnitude as well as the sign depending on the details of the device structure (Table 2 of ref 11 and refs 14, 15 ). Therefore, this indicates that Im­(*g* ^↑↓^) is sizable and the modulation of the gyromagnetic ratio due to Im­(*g* ^↑↓^) in spin pumping experiments cannot be ignored. So far, there are no experimental reports for the detection of the modulation of the *g*-factor by spin pumping in FM/HM bilayers, possibly because it is hard to detect the ferromagnetic resonance (FMR) of ultrathin FM with significantly enhanced α_eff_.

Recently, a linear *t* ^–1^–*g*_eff_ behavior is found in a series of ultrathin Fe films grown on GaAs(001) substrate where a quasi two-dimensional electron gas (2DEG) forms at the Fe/GaAs interface.^16^ This indicates that spin pumping leads to the modulation of *g*_eff_ (Supplementary Note 2). Surprisingly, the magnitude of Im­(*g* ^↑↓^) is about one order larger than Re­(*g* ^↑↓^), which indicates that the spin transport in the *z*-direction of the FM/2DEG system differs significantly from the diffusive spin transport in FM/HM bilayers with finite spin-diffusion lengths.^4–8^ Although the mixing conductance in FM/2DEG bilayers has not been described theoretically, the preliminary results suggest that FM/2DEG could be ideal systems to study the modification of *g*_eff_ by spin pumping. In this work, we show that spin pumping can also lead to a significant modulation of the *g*-factor in another FM/2DEG system, i.e., the 2DEG at the AlO_*x*_/SrTiO_3_ interface with strong SOI. Specifically, we observe an anisotropic *g*-factor with 2-fold symmetry below the onset temperature of the 2DEG and a back gate-voltage even tunes this anisotropy by changing the Fermi level of the 2DEG.

To observe the modification of the Landé *g-*factor, we revisit the spin pumping experiments in Py/Al/STO multilayers.^17,18^ Previous studies have demonstrated that depositing ultrathin Al on STO extracts oxygen from insulating Ti^4+^ ions in the STO substrate, creating electron-rich Ti^3+^ and/or Ti^2+^ states.^19^ This process oxidizes the Al layer into AlO_*x*_ (hereafter referred to as Py/AlO_*x*_/STO), while simultaneously forming a 2DEG at the AlO_*x*_/STO interface, more accurately, within the first atomic planes of the STO surface. Angle-resolved photoemission spectroscopy (ARPES) studies confirm that even a 0.2 nm Al layer suffices to create this 2DEG.^19^ Consequently, the AlO_*x*_/STO system has emerged as a versatile platform for studying magnetism, superconductivity, and SOI, akin to the well-known 2DEG at the LaAlO_3_/STO interface.^20^ The surface-proximity of STO-based 2DEG makes it particularly suitable for spin injection experiments such as spin pumping. Recent experiments have revealed an exceptionally large spin-to-charge conversion efficiency at the AlO_*x*_/STO,^17,18^ the LAO/STO,^21^ and LaTiO_3+δ_/STO interfaces,^22^ which is ascribed to the large SOI and the enhanced momentum relaxation time in the 2DEG. Subsequent calculations by Johansson et al. further suggest that both spin-Rashba effect and orbital Rashba effect (ORE) contribute to the conversion, with ORE potentially dominating by at least an order of magnitude.^23^ The existence of ORE has been demonstrated by the measurement of the anisotropic spin Seebeck voltage in Py/LAO/STO multilayers, in which the anisotropic spin Seebeck voltage is interpreted through the interplay between the anisotropic spin-to-charge and the isotropic orbit-to-charge conversion mechanisms.^24^

We grow AlO_*x*_ (6 nm)/Py (Ni_80_Fe_20_, 6 nm)/Al (1 nm) multilayers on STO (001) substrate by molecular-beam-epitaxy (Supplementary Note 1). Prior to growth, the substrate is heated in a vacuum up to 700 °C for 10 min. To minimize interdiffusion and especially agglomeration of Al, the substrate temperature is further cooled below −140 °C when we deposit the Al and Py layers. Finally, a 6 nm AlO_*x*_ capping layer is deposited to avoid oxidation of Py. High-resolution transmission electron microscopy (HRTEM) (Figure 1a) and high-angle annular dark field scanning transmission electron microscopy (HAADF-STEM) (Figure 1b) images show that the Py/Al and Al/STO interfaces are sharp and flat, with no significant interdiffusion of atoms. The STO surface is terminated by Ti atoms, and the thickness of the AlO_*x*_ layer is estimated to be ∼ 1 ± 0.1 nm, slightly thinner than that expected from 1 nm Al deposition and its volume expansion by oxidation. The energy-dispersive X-ray (EDX) images (Figure 1c) show that oxygen has extended to the Al layer, and thus Al is oxidized to AlO_*x*_.

**Figure 1:**
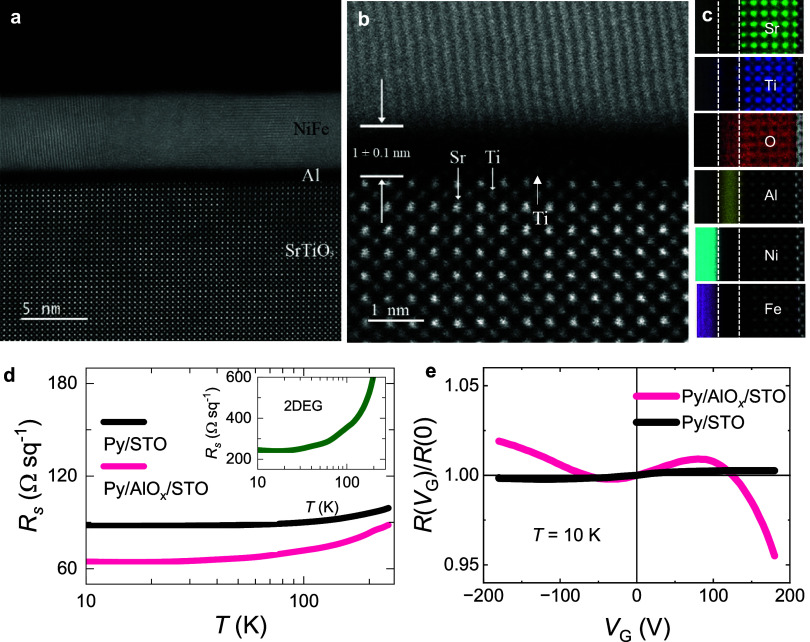
(a) HRTEM image of the Py/AlO_*x*_/STO multilayers. (b) Magnified view of the Py/AlO_*x*_ and AlO_*x*_/STO interfaces. (c) HAADF-STEM image and EDX of each element. (d) Temperature dependence of the sheet resistance of Py/AlO_*x*_/STO (red line) and Py/STO (black line); the resistance of AlO_*x*_/STO 2DEG is shown in the inset by the green line. (e) Gate-voltage *V*_G_ dependence of *R*(*V*_G_)/*R*(0) for Py/STO and Py/AlO_*x*_/STO measured at 10 K.

Figure 1d shows the temperature (*T*) dependence of the resistance for Py/AlO_*x*_/STO and Py/STO, in which a drop of the resistance is observed around 100 K for Py/AlO_*x*_/STO. The extracted resistance of the 2DEG by the parallel resistance model shows the onset of the 2DEG around 100 K. Figure 1e presents the resistance measured by back gate-voltage *V*_G_ tuning, showing that the resistance of Py/STO is almost independent of *V*_G_, while for Py/AlO_*x*_/STO a sizable modulation is observed. These results further confirm the presence of a 2DEG for Py/AlO_*x*_/STO. Note that an unusual *V*_G_-dependence of resistance is observed for −50 V < *V*_G_ < +80 V for both samples; the resistance slightly increases as *V*_G_ increases. The reason for this reproducible behavior is unclear to us, and it has not been reported before.

For FMR measurements, we fabricate 7 μm × 320 μm Py/AlO_*x*_/STO stripes integrated into the gap between the signal and ground lines of a coplanar waveguide (CPW) (Figure 2b). Under microwave excitation, the dc voltage spectrum *V*(*H*) induced by FMR is measured while sweeping the magnetic-field *H*. Figure 2c shows typical *V*(*H*) trace measured at the magnetic-field angle φ_H_ = 90°, at a microwave frequency *f* of 8 GHz, and *T* of 10 K. The spectrum is well-fitted by a combination of a symmetric (*L*_sym_ = Δ*H* ^2^/[4­(*H* *–* *H*_R_)^2^ + Δ*H* ^2^]) and an antisymmetric (*L*_a‑sym_ = −4Δ*H*(*H*–*H*_R_)/[4­(*H*–*H*_R_)^2^ + Δ*H* ^2^]) Lorentzian, i.e., *V*–*V*_offset_ = *V*_sym_ *L*_sym_ + *V*_a‑sym_ *L*_a‑sym_. Here *H*_R_ is the resonance field, Δ*H* the full width at half-maximum, *V*_offset_ the offset voltage, and *V*_sym_ (*V*_a‑sym_) the symmetric (antisymmetric) component of the dc voltage. Here, *V*_a‑sym_ arises solely from the anisotropic magneto-resistance (AMR) effect of Py, while *V*_sym_ contains additional contributions from the spin pumping voltage induced by the inverse spin Rashba-Edelstein effect at the AlO_*x*_/STO interface. Note that since the direction of detection of the d.c. voltage is parallel to the microwave current, the dynamic tunneling anisotropic magneto-resistance effect^25^ and the dynamic Hanle effect,^26^ which are detectable in a geometry transverse to the microwave current, cannot contribute to the signal. Before, we have shown that, for Si^27^ and GaAs^28^ substrates with low dielectric constant, the out-of-plane Oersted field induced by the rf current flowing in the CPW is the dominating excitation for magnetization dynamics, and it is possible to separate spin pumping voltages from AMR, while for STO substrate with large dielectric constant (∼20000 at 10 K), this separation becomes impossible for most of the measured gate-voltages because the in-plane excitation induced by the shunting current flowing in STO dominates (ref 29 and Supplementary Note 3). Below, we primarily focus on the discussion of *H*_R_, which has largely been overlooked in spin pumping experiments.

**Figure 2:**
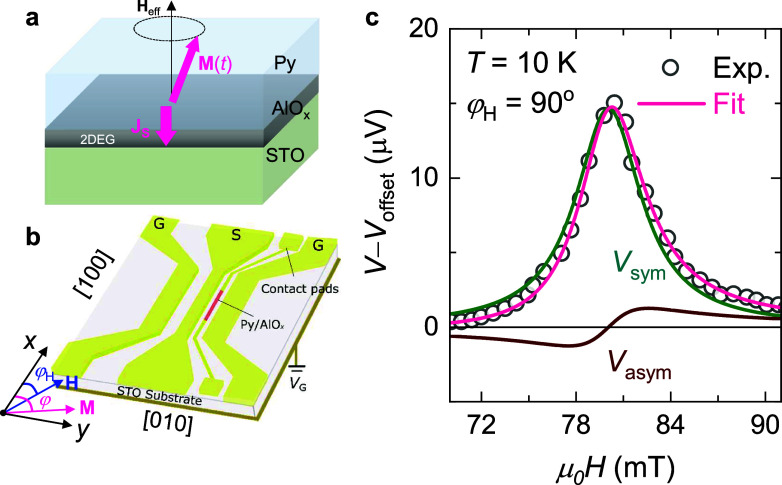
(a) Schematic of spin pumping from Py into the AlO_*x*_/STO 2DEG. (b) Schematic of spin pumping device. φ_H_ (φ) is the angle of the magnetic-field **H** (the magnetization **M**). (c) Typical dc voltage spectra measured at *T* = 10 K, *f* = 8 GHz, and φ_H_ = 90°. The solids are the Lorentz fits.

Figure 3a shows the φ_H_-dependence of *H*_R_ measured at *f* = 8 GHz for *T* = 120 K, 80K, 50, and 20 K. At 120 K, the *H*_R_ trace exhibits a clear uniaxial anisotropy with the minimum values (*H*_R_^*min*^ ∼ 74.2 mT) occurring when **H** is parallel to the stripe (φ_H_ = 0° and 180°, easy axis) and with maximum values (*H*_R_^max^ ∼ 76.6 mT) occurring when **H** is perpendicular to the stripe (φ_H_ = 90° and 270°, hard axis). This 2-fold symmetry of *H*_R_ is well-described by the resonance condition,^30^

4 $${\left(\frac{2\pi f}{\gamma }\right)}^{2}=\mu_{0}^{2}H_{1}H_{2}$$

where *H*_1_ = *H*_R_ cos (φ–φ_H_) + *H*_K_ + *H*_U_ cos ^2^φ, *H*_2_ = *H*_R_ cos (φ–φ_H_) + *H*_U_ cos 2φ, φ the angle of **M**, *H*_K_ the effective demagnetization anisotropy field, and *H*_U_ the uniaxial shape anisotropy field. From the fits, the values of *g*, *H*_K_, and *H*_U_ are respectively determined to be 2.1, 914, and 0.87 mT. The magnitude of *H*_K_ matches the saturation magnetization of Py, i.e., *H*_K_ ∼ *N*_*zz*_ *M*, where *N*_*zz*_ is the out-of-plane demagnetizing factor, confirming its dominant origin from thin-film demagnetization with negligible perpendicular anisotropy at the Py/AlO_*x*_ interface. Similarly, *H*_U_ quantitatively matches the anisotropy field induced by the shape of the Py stripe, i.e., *H*_U_ ∼ *N*_*yy*_ *M*, where *N*_*yy*_ is the in-plane demagnetizing factor perpendicular to the stripe (Supplementary Note 4).

**Figure 3:**
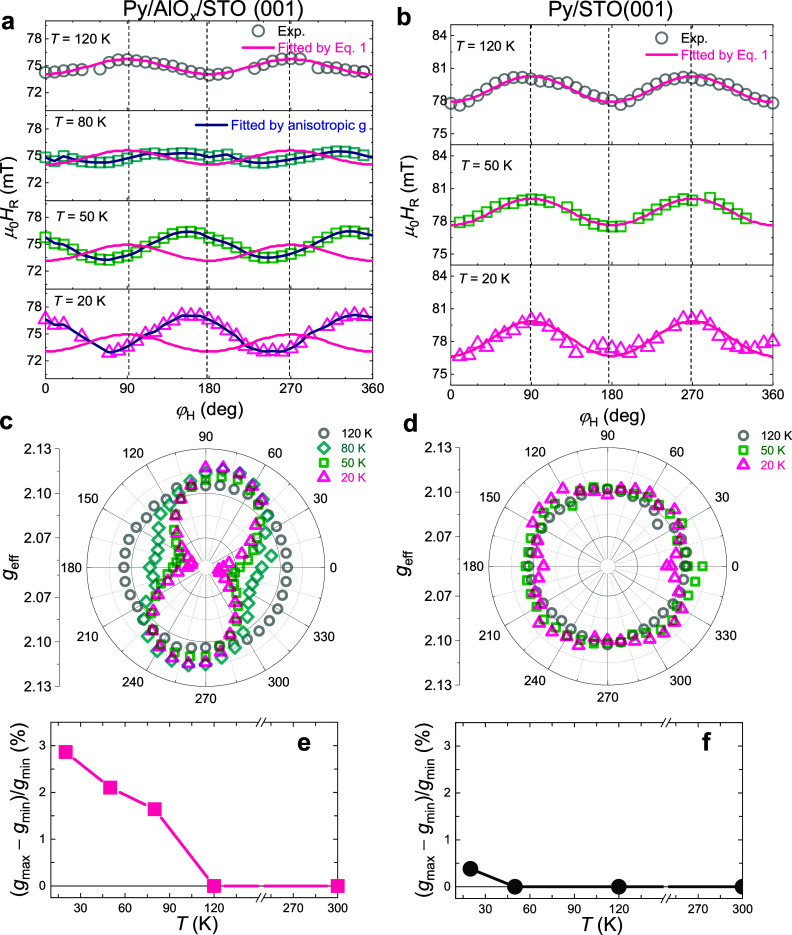
(a) φ_H_-dependence of the resonance field *H*_R_ of the Py/AlO_*x*_/STO sample measured at *f* = 8 GHz and *T* = 120 K, 80 K, 50 K, and 20 K. (b) φ_H_-dependence of *H*_R_ of the Py/STO sample measured at *f* = 8 GHz and *T* = 120 K, 50 K, and 20 K. The pink lines in (a) and (b) are calculated by eq 4 using the shape anisotropy of the stripe, and the blue lines in (a) are calculated by considering anisotropic *g*-factor. (c) Polar plot of the anisotropic Landé *g*-factors for the Py/AlO_*x*_/STO samples at different temperatures. (d) Polar plot of the *g*-factors for the Py/STO samples at different temperatures. (e) and (f) *T*-dependence of the *g*-anisotropy, (*g* ^max^–*g* ^*min*^)/*g* ^*min*^, for Py/AlO_*x*_/STO and for Py/STO.

As *T* is decreased to 80 K (i.e., below the onset temperature for the 2DEG), the angular trace of *H*_R_ differs significantly from that obtained at 120 K: *H*_R_^*min*^ now appears at 75° (255°) and *H*_R_^max^ at 165° (345°). This phase shift persists down to 20 K with an enhanced *H*_R_^max^-*H*_R_^*min*^ value of 4 mT. Obviously, the unusual angular trace of *H*_R_ at lower temperatures, which has been reproduced by several other stripe- and full-film- samples (Supplementary Note 5), cannot be explained by considering the uniaxial shape anisotropy of the stripe (pink lines). To exclude any possibility that the shift of *H*_R_ arises from an unknown magnetic anisotropy induced by the STO substrate, we additionally prepared a Py (6 nm)/STO bilayer and performed similar measurements. As shown in Figure 3b, no shift of *H*_R_ is observed for all measured temperatures, i.e., *H*_R_^*min*^ (*H*_R_^max^) appears for φ_H_ = 0° and 180° (φ_H_ = 90° and 270°), and all the *H*_R_-traces can be well-fitted by eq 4 using the shape anisotropy of the stripe rather than by additional magnetic anisotropies induced by the STO substrate. Moreover, all the samples studied here are capped by AlO_*x*_, and thus any unknown effect from the Py/AlO_*x*_ interface can also be excluded.

Therefore, the observed *H*_R_-trace phase shift in the Py/AlO_*x*_/STO device must be interpreted in terms of an anisotropic Landé *g*-factor induced by spin pumping. By measuring the frequency-dependence of *H*_R_ at fixed φ_H_ angles (Supplementary Note 6) and fitting with eq 4 (fixing *H*_K_ and *H*_U_ values while varying *g*), a clear 2-fold symmetry of the *g*-factor is obtained at *T* = 50 K and 20 K. The minimum *g* ^*min*^ appears at 165° (345°) while the maximum *g* ^max^ appears at 75° (255°) (Figure 3c), and the relative anisotropy (*g* ^max^–*g* ^*min*^)/*g* ^*min*^ increases as *T* decreases (Figure 3e). In contrast, the Py/STO control sample shows nearly isotropic *g*-values at *T* = 120 and 50 K (Figure 3d), and the relative anisotropy (*g* ^max^–*g* ^*min*^)/*g* ^*min*^ shows weak increases as *T* decreases (Figure 3f). For *T* = 20 K, a weak 2-fold anisotropy is observed, which could be related to the emergence of a weaker 2DEG at the Py/STO interface^31^ (one may also note a weak 4-fold symmetry in Figure 3d. However, due to its weakness, it is difficult to clearly identify.).

To verify that the angular momentum absorption by the 2DEG at the AlO_*x*_/STO interface indeed plays a role for the shift of *H*_R_, we insert a 4 nm Pt layer between Py and STO as an isotropic spin sink and perform the same measurements as the Py/AlO_*x*_/STO and the Py/STO samples. As shown in Figure S9, the Pt insertion completely suppresses the unusual *H*_R_-shift, and all the *H*_R_ traces can be interpreted by the uniaxial shape anisotropy of the Py stripe with an isotropic *g*-factor. This confirms again that the emergence of the anisotropic *g*-factor in Py/AlO_*x*_/STO is related to spin pumping into 2DEG and is not from any unknown magnetic anisotropies induced by the substrate.

The exceptionally large dielectric constant^32^ of STO combined with the 2DEG’s low carrier density enables effective Fermi level tuning via back gate-voltage, as evidenced by the sizable modulation of the resistance (Figure 1e). Here, we address the influence of *V*_G_ on *H*_R_ and thus on the anisotropic *g*-factor. Figure 4a-4c shows the *V*_G_-dependence of *H*_R_ measured at *f* = 7 GHz and *T* = 10 K for different magnetic-field angles. For φ_H_ = 170° and 350° (Figure 4a), *H*_R_ decreases with *V*_G_ and the modulation of *H*_R_ is about −2 mT; for φ_H_ = 40° and 220° (Figure 4b), *H*_R_ is almost independent of *V*_G_; however, for φ_H_ = 60° and 240° (Figure 4c), *H*_R_ increases with *V*_G_ and the modulation of *H*_R_ is about +2 mT. This φ_H_-dependence of the *H*_R_ response indicates that the modulation of *H*_R_ does not originate from the change of magnetic anisotropies of Py by *V*_G_ via the magnetoelectric effect^33^ (Supplementary Note 11), since: I) the electric-field primarily affects the 2DEG rather than the Py film, and the magnetic properties of Py can be hardly modified by *V*_G_ because of its high carrier concentration (∼10^23^ cm^–3^), II) If there were a modulation of *H*_K_ and/or *H*_U_ by *V*_G_, the dependence of *H*_R_ on *V*_G_ should be independent of φ_H_.

**Figure 4:**
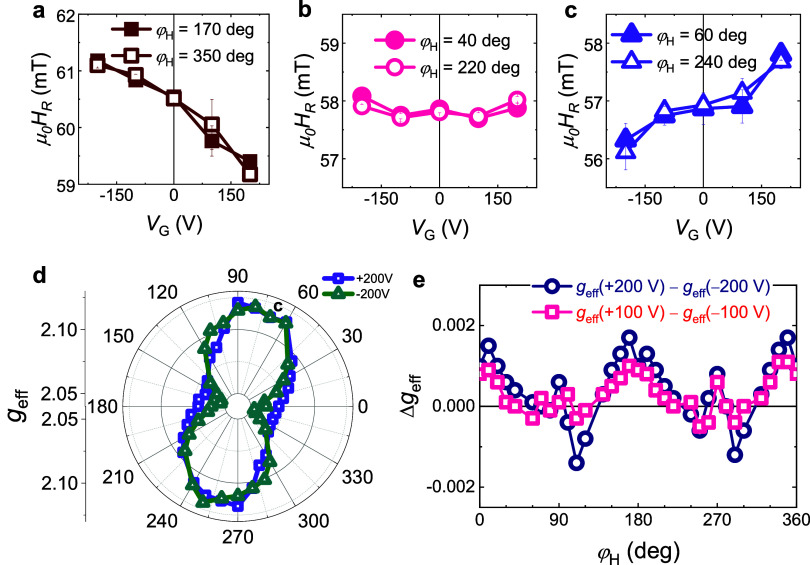
(a), (b), and (c): *V*_G_-dependence of *H*_R_ for Py/AlO_*x*_/STO measured at *f* = 7 GHz and *T* = 10 K for φ_H_ = 170°/350°, φ_H_ = 40°/220°, and φ_H_ = 60°/240°. (d) Polar plot of the anisotropic *g*-factor values for *V*_G_ = −200 V and +200 V. (f). φ_H_-dependence of Δ*g*, where Δ*g*(±*V*_G_) = *g*(+*V*_G_)−*g*(−*V*_G_).

Figure 4d summarizes the φ_H_ *-*dependent *g*-value anisotropy for *V*_G_ = +200 V and +200 V. The *g*-factor shows an anisotropic modification, i.e., for φ_H_ = 170° and 350° *g*(+200 V) < *g*(+200 V) holds; for φ_H_ = 40° and 220° *g*(+200 V) ∼ *g*(−200 V) holds; while for φ_H_ = 60° and 240°, *g*(+200 V) > *g*(+200 V) holds. The gate-tunable anisotropy becomes more pronounced when Δ*g*(±*V*_G_) = *g*(+*V*_G_)-*g*(−*V*_G_). As shown in Figure 4e, |Δ*g*(±200 V)| > |Δ*g*(±100 V)| holds, and Δ*g* changes sign several times when φ_H_ varies from 0° to 360°, indicating that Δ*g* is also anisotropic. Control measurements on Py/Pt/STO and Py/STO samples (Supplementary Notes 9 and 10) show no modulation for the former and significantly weaker modulation for the latter (Figure 5), confirming that the anisotropic *g*-factor tuning in the Py/AlO_*x*_/STO sample originates from the modulation of the Fermi level of the 2DEG.

**Figure 5:**
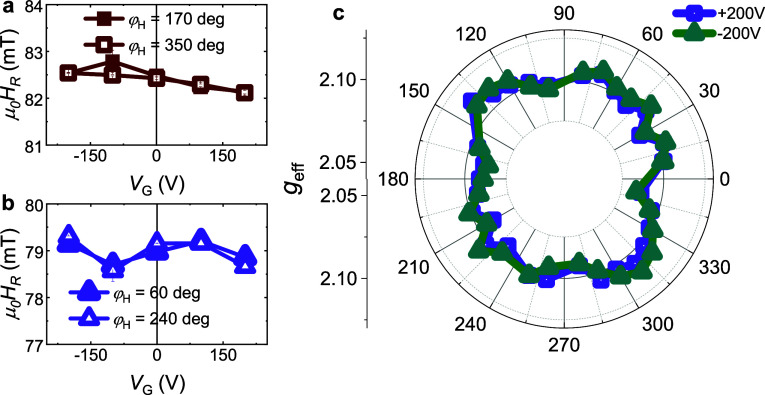
(a) and (b): *V*_G_-dependence of *H*_R_ for Py/STO measured at *f* = 8 GHz and *T* = 10 K for φ_H_ = 170°/350° and φ_H_ = 60°/240°. (c) Polar plot of the corresponding *g*-factor values for *V*_G_ = −200 V and +200 V.

Under FMR, the magnetization precession in the polycrystalline Py layer injects spin currents into the 2DEG (Figure 2a), and the injected spin currents are isotropic. However, if the spin sink is anisotropic, this can lead to the emergence of an anisotropic *g*-factor. Previous theoretical and experimental studies have shown that the anisotropic spin textures for the STO-based 2DEG show a 4-fold symmetry.^23,24^ Therefore, this should lead to a 4-fold symmetry of the anisotropic *g*-factor which cannot explain the 2-fold symmetry as observed in the experiments. On the other hand, the pristine STO is an insulating cubic perovskite with a lattice constant of 3.905 Å when *T* > 105 K. Below 105 K, STO undergoes the cubic-to-tetragonal structural phase transition,^34,35^ accompanied by a broken in-plane rotation symmetry^36^ with *a* < *b* < *c*, where *a* and *b* are the lattice constants in the (100) plane of the tetragonal structure, and *c* is the lattice constant along [001]. A *local* scanning tunneling microscopy characterization reveals that this broken rotational symmetry is more significant and an enhanced *b*/*a* up to 1.19.^37^ Over a wide range of surface oxygen vacancies, the broken rotational symmetry leads to weakly unidirectional electronic states, mostly aligned along the [110]-direction. Moreover, ARPES measurements can also not exclude this weak anisotropy.^17,19,38^ Since the mixing conductance is expressed in terms of spin-dependent reflection and transmission coefficients for electronic states at the interface^2,4^ and the reflection and transmission coefficients should be proportional to the density of states at Fermi level, the emergence of 2-fold anisotropic *g*-factor could be induced by the anisotropic density of states at the 2DEG. Therefore, we infer that the unidirectional electronic states may be responsible for the observed 2-fold anisotropic *g*-factor at low temperatures, where the angular momentum absorption efficiency is enhanced due to an increase of the momentum relaxation time. On the other hand, the broken rotational symmetry may induce Dresselhaus SOI, and the interplay of Rashba and Dresselhaus terms could account for our observations. Additional experimental and theoretical work is needed to confirm the Dresselhaus SOI in STO systems.

It is known that one of the hallmarks of spin pumping measurements in heavy metal/FM bilayers is the enhancement of the Gilbert damping value compared to that of the single FM.^2,3^ In principle, if the spin sink has an anisotropic electronic texture at the Fermi level, both an anisotropic damping^39,40^ and an anisotropic *g*-factor should be observed. However, our results show that, within experimental accuracy, the damping is almost isotropic and independent of *V*_G_ (Supplementary Notes 8, 9, and 10). One reason for this is the coexistence of orbital pumping.^24,41–43^ Previous studies have shown that the magnitude of spin moment for AlO_*x*_/STO 2DEG is much smaller than the orbital moment,^23,24^ and this leads to less damping enhancement from the loss of spin angular momentum of Py and an insensitivity to *V*_G_. Moreover, the relatively small orbit-to-spin magnetization ratio in Py limits the orbital-current flow into the 2DEG, reducing the orbital pumping contribution to the damping.^44^ However, because of the significant shunting current in STO, it is not possible to accurately separate the orbital pumping voltage from the symmetric AMR effect (Supplementary Note 3).

Our results show a clear gyromagnetic ratio modulation in FMR pumping experiments. The effect has been theoretically predicted for a long time,^2^ but the experimental detection was still missing.^16^ We note that a quantitative understanding of the anisotropic modulation by gate-voltage, which could arise from the competition of various Fermi-energy-dependent effects, e.g., the anisotropic electron density or the interplay between spin angular momentum and orbital angular momentum absorption, is still elusive. We also note that a sign reversal of the Rashba coefficient has been observed in several STO-based 2DEGs,^17,18,21^ ^,^ ^22,24^ and it is unclear for us how to connect the *g*-factor modulation with the Rashba-SOI. The discrepancy between the *g*-factor modulation and the Rashba coefficient modulation might arise from the fact that the *g*-factor is related to Im (***g*** ^↑↓^) while the extracted Rashba coefficient is related to Re (***g*** ^↑↓^). Qualitative and quantitative theory are still missing so far. The emergence of the anisotropic *g*-factor results from spin pumping in the presence of an anisotropic electronic distribution. The modulation of the gyromagnetic ratio in FMR pumping experiments highlights its potential as a probe for the imaginary part of mixing conductance in an FM/2DEG system. The modulation is not limited through the spin-charge and orbital-charge conversion processes. Their inverse processes, i.e., current-induced angular momentum accumulation, can also lead to a modulation of the *g*-factor and magnetism.^45^ These findings bridge a critical gap between theory and experiment, offering new avenues to manipulate magnetic properties in spin and orbitronic devices.

## Supplementary Material



## Notes

Methods, Mixing conductance in Fe/GaAs quasi two-dimensional system, Spin pumping voltage in Py/AlOx/STO multilayers, Quantifying the shape anisotropy of the Py stripe, Angular dependence of *H*_R_ for other Py/AlOx/STO samples, Frequency dependence of *H*_R_ in Py/AlOx/STO and Py/STO devices, Isotropic g-factor in Py/Pt/STO, Angular- and temperature-dependence of damping in Py/AlOx/STO, Py/STO and Py/Pt/STO, *V*_G_-dependence of damping in Py/AlOx/STO, *V*_G_-dependence of resonance field and damping in Py/Pt/STO and Py/STO, Excluding the charge mediated magnetoelectric coupling effect in gate modulation of measurement (PDF)
