# Supplementary material for: Electric-Field Tunable Anisotropic g‑Factor Induced by Spin Pumping
Source: Nano Lett. 2026 Feb 20;26(8):2853–60. doi: 10.1021/acs.nanolett.5c05536 (PMC12964550; doi:10.1021/acs.nanolett.5c05536)
Supplement: Supplementary file 1 [file nl5c05536_si_001.pdf]

Supplementary Material for

Electric-field tunable anisotropic *g*-factor induced by spin pumping

Jian Shao[1,2], Matthias Kronseder[3], Jianping Guo[2,7], Maximilian Mangold[2,7], Dong Pan[4], Thomas N. G. Meier[2,7], Weiwei Zhao[1*], Jianhua Zhao[4,5], Christian H. Back[2,6,7], and Lin Chen[2,7*]

[1]*Sauvage Laboratory for Smart Materials, School of Integrated Circuit, Harbin Institute of Technology, Shenzhen 518055, China*

[2]*Department of Physics, Technical University of Munich, 85748 Garching b. Munich, Germany*

[3]*Institute of Experimental and Applied Physics, University of Regensburg, 93049 Regensburg, Germany*

[4]*State Key Laboratory of Semiconductor Physics and Chip Technologies, Institute of Semiconductors, Chinese Academy of Science, Beijing 100083, China*

[5]*National Key Laboratory of Spintronics, Hangzhou International Innovation Institute, Beihang University, 11115 Hangzhou, China*

[6]*Munich Center for Quantum Science and Technology (MCQST), 80799 Munich, Germany*

[7]*Center for Quantum Engineering (ZQE), Technical University of Munich, 85748 Garching b. Munich, Germany*

Table of Contents:

1. Methods
2. Mixing conductance in Fe/GaAs quasi two-dimensional system
3. Spin pumping voltage in Py/AlO$_x$/STO multilayers
4. Quantifying the shape anisotropy of the Py stripe
5. Angular dependence of H_R for other Py/AlO$_x$/STO samples
6. Frequency dependence of H_R in Py/AlO$_x$/STO and Py/STO devices
7. Isotropic *g*-factor in Py/Pt/STO
8. Angular- and temperature-dependence of damping in Py/AlO$_x$/STO, Py/STO and Py/Pt/STO
9. V_G-dependence of damping in Py/AlO$_x$/STO
10. V_G-dependence of resonance field and damping in Py/Pt/STO and Py/STO
11. Excluding the charge mediated magnetoelectric coupling effect in gate modulation of measurement

Supplementary Note 1: Methods

Sample preparation.

The preparation of the $SrTiO_3(001)$ substrates (CrysTec GmbH, Germany) included heating in vacuum to 700 °C for 10 min., followed by cooling the substrate to temperatures lower than −140 °C before depositing the Al and all subsequent layers in a chamber dedicated to molecular-beam-epitaxy and a base pressure of lower than 1×10^{-10} mbar. A 6 nm AlO_x layer is used to cap the Py layer to avoid oxidation in air.

Sample characterization.

Structural characterization was performed by transmission electron microscopy system using a JEOL ARM300F operated at 300 kV. The cross-sectional lamellas were prepared by a double beam focused ion beam system, FEI Helios G4 CX, operated at 10 kV. The chemical components of the specimen were measured by energy dispersive X-ray spectroscopy during high-angle annular dark field scanning transmission electron microscopy observations.

Device fabrication.

For the spin pumping device, a coplanar waveguide (CPW) was fabricated directly onto the sample using a mask-free writer followed by evaporation of 5 nm Ti and 100 nm Au. The width of the signal line and the gap were 50 μm and 30 μm, respectively. The strip with dimensions of 7 μm in width and 320 μm in length was patterned in the gap region via negative photoresist and Ar-etching, two contact pads were subsequently connected to the strip to detect the dc voltage. The Hall bar was patterned with 20 μm width and 100 μm length. During the fabrication, the baking temperature was kept below 90 °C to protect the sample. After fabrication, all devices were checked to ensure the etched areas remained electrically insulating thereby avoiding any conductive paths that could result from over-etching.

Measurements.

For spin pumping measurements, microwave currents with frequencies ranging from 6-20 GHz and input power ranging between 10 to 25 dBm is generated by a Rohde & Schwarz SMA100B device. The d.c. voltage was measured using an Agilent 34420A nanovoltmeter. For the Hall measurement, the R_{xy} and R_{xx} resistances were measured using a standard four-terminal configuration with a direct current of 10 μA. All the measurements were conducted in a Cryo 3D system,

which allows for temperature control from 300 K down to 10 K and three-dimensional magnetic field rotation (*x*-*y*-*z* axes). Gate-voltage sweeps were performed using a Keithley 2450 sourcemeter, with voltages ranging from +200 V to −200 V. To avoid hysteresis effects associated with the $SrTiO_3$ substrate, each measurement sequence was preceded by a full gate-voltage sweep from +200 V to −200 V and then back to +200 V. The gate leakage current was maintained below 10 nA throughout the measurements, which is too small to change the magnetic properties of Py by Joule heating.

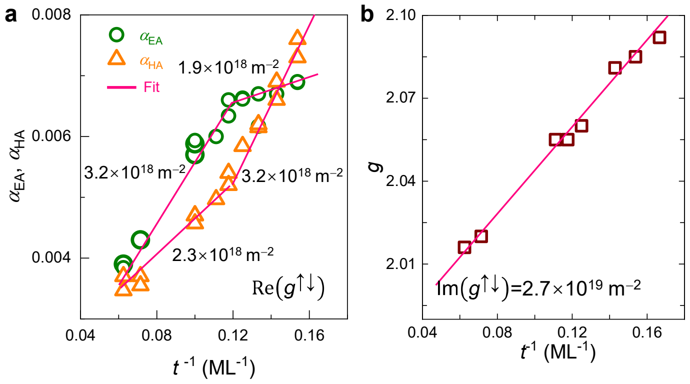

Fig. S1. (**a**) Inverse Fe thickness (t^{-1}) dependence of the damping constant along the easy (α_{EA}) and hard (α_{HA}) axes of Fe/GaAs bilayers, where a quasi-two-dimensional electron gas forms at the interface. The solid lines are fits to equation (2) and the obtained Re($g_{eff}^{\uparrow\downarrow}$) value for each regime is indicated in the figure. (**b**) t^{-1}-dependence of the gyro-magnetic ratio, and the solid line is a fit to equation (3). The obtained Im($g_{eff}^{\uparrow\downarrow}$) is about one order larger than Re($g_{eff}^{\uparrow\downarrow}$).

Supplementary Note 2: Mixing conductance in Fe/GaAs quasi two-dimensional system

Figs. S1a and S1b show the damping parameters and the Landé g-factor as a function of inverse Fe thickness (t^{-1}) for the Fe/GaAs system, where a quasi two-dimensional electron gas forms at the interface (data taken from Ref. 13). The magnitude of α_{EA} and α_{HA} differs because of the emergence of anisotropic damping. Both α_{EA} and α_{HA} scale linearly with t^{-1} but with different slopes in different regimes, suggesting the emerging anisotropic effective spin mixing conductance. The magnitude of Re($g_{eff}^{\uparrow\downarrow}$) in each regime is calculated using equation 2, which is of the order of 10^{18} m^{-2}. Fig. S1b shows the g value of the same sample series and a linear dependence of g on t^{-1} is observed. By fitting the trace by equation 3, the magnitude of Im($g_{eff}^{\uparrow\downarrow}$) is determined to be 2.7×10^{19} m^{-2}, which is about one order larger than Re($g_{eff}^{\uparrow\downarrow}$). As far as we

know, this is the first report of the modulation of the *g*-factor by spin pumping (note that the existence of spin pumping has also been confirmed by the measurements of the inverse spin galvanic effect[23]). Surprisingly, $\mathrm{Im}(g_{\mathrm{eff}}^{\uparrow\downarrow})$ dominatesover $\mathrm{Re}(g_{\mathrm{eff}}^{\uparrow\downarrow})$, indicating that the spin transport in a FM/2DEG system differs from that in HM/FM bi-layers. The possible reason is that the spin transport in FM/HM bilayers is diffusive with finite spin diffusion length, whereas in FM/2DEG system, the interface governs spin dephasing. This implies that FM/2D heterostructures could be an ideal system for the study of the modulation of the *g*-factor by spin pumping. Future theory on this topic is needed to fully understand the larger $\mathrm{Im}(g_{\mathrm{eff}}^{\uparrow\downarrow})$ value as well as the anisotropic $\mathrm{Re}(g_{\mathrm{eff}}^{\uparrow\downarrow})$.

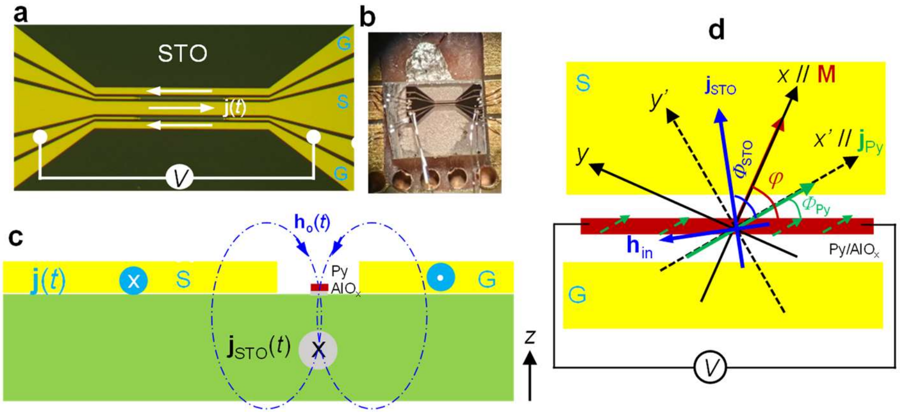

Fig. S2. (**a**) Optical microscopy image of the coplanar wave guide (CPW) with signal line (S) and ground lines (G) on top of the STO substrate. The Py/AlO_x/STO stripes are integrated into the gap between signal and ground lines, and the dc voltage *V* is detected between the two ends of the stripe. The white arrows represent the rf current flow in signal and ground lines. (**b**) Optical microscopy image of the device with sample holder. (**c**) Schematic of the out-of-plane excitation field h_o induced by the rf currents flowing in S and G. The high relative permittivity and dielectric loss of STO cause a significant shunting current $\mathbf{j}_{\mathrm{STO}}$ in STO. (**d**) Schematics of the coordinate systems for separating the pumping voltage from rectifying effects. The induced microwave current in Py $\mathbf{j}_{\mathrm{Py}}$ is assumed to be along the *x*' direction, which forms an angle Φ_{Py} with respect to the Py/AlO_x/STO stripe. The magnetization **M** is along the *x*-axis, and φ is magnetization angle. $\mathbf{j}_{\mathrm{STO}}$ is the shunting current in STO flowing underneath Py, which forms an angle Φ_{STO} with respect to the stripe, and $\mathbf{h}_{\mathrm{in}}$ is the in-plane Oersted field induced by $\mathbf{j}_{\mathrm{STO}}$. In the experiment, the magnitudes of Φ_{Py} and Φ_{STO}, which depend on the microwave frequency and the gate-voltage can be quantified experimentally.

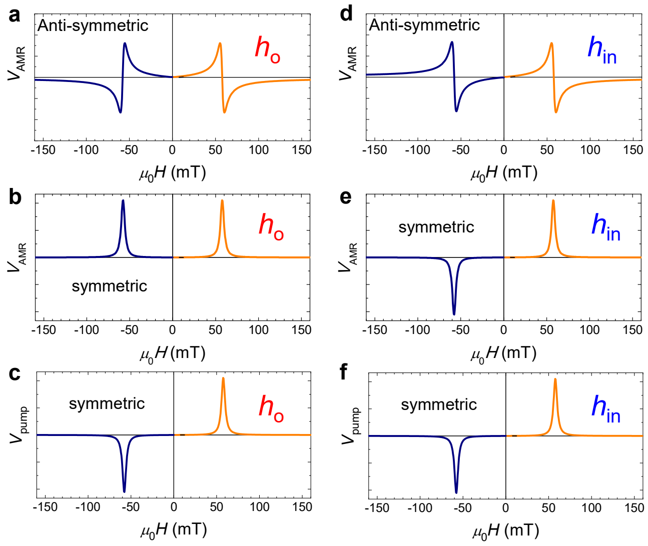

Fig. S3. Symmetry of the dc voltage for the anti-symmetric AMR voltage V_{AMR}^{A} (**a**), the symmetric AMR voltage V_{AMR}^{S} (**b**) and the symmetric spin pumping voltage V_{Pump}^{S} (**c**) induced by out-of-plane excitation h_o. (**d**), (**e**), (**f**): the same as (**a**), (**b**), (**c**) but for in-plane excitation h_{in}.

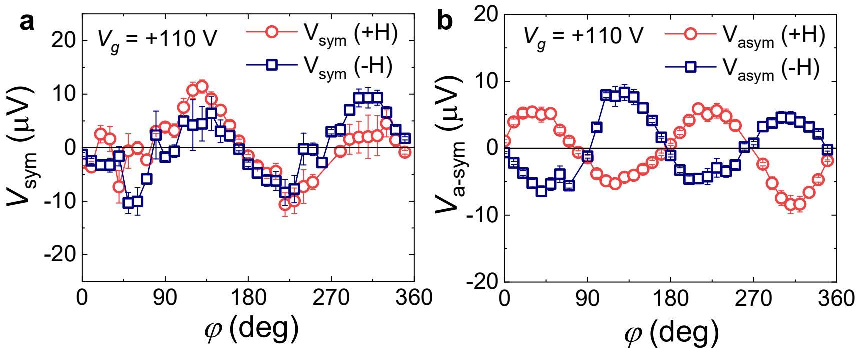

Fig. S4. (**a**) φ-dependence of $V_{sym}(+H)$ and $V_{sym}(-H)$ for Py/AlO$_x$/STO sample measured at f = 8 GHz, T = 10 K and V_G = +110 V. (**a**) φ-dependence of $V_{a\text{-}sym}(+H)$ and $V_{a\text{-}sym}(-H)$.

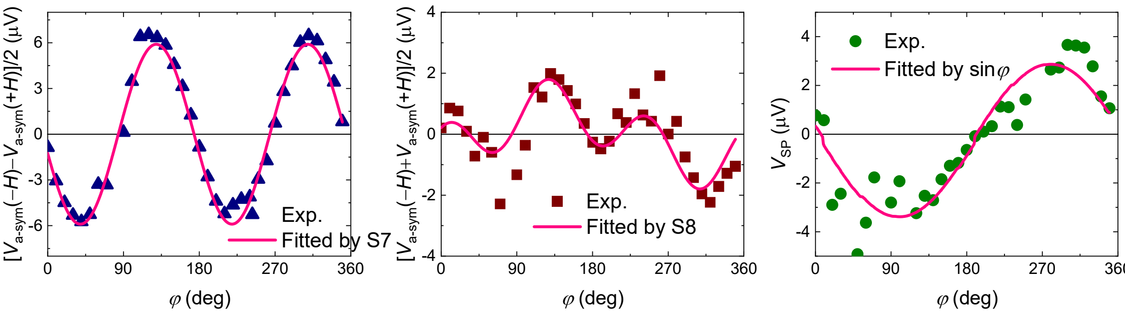

Fig. S5. (**a**) φ-dependence of $[V_{a\text{-}sym}(-H) - V_{a\text{-}sym}(+H)]/2$ obtained for V_G = +110 V. The solid line is the fit to equation S7

and Φ_{Py} is determined. (**b**) φ-dependence of $[V_{a\text{-}sym}(-H) + V_{a\text{-}sym}(+H)]/2$. The solid line is the fit to equation S8 and Φ_{STO} is determined. (**c**) φ-dependence of spin pumping voltage $V_{pump} = [V_{sym}(-H) - V_{sym}(+H)]/2$, and the solid line is fitted by $\sin\varphi$. These results prove that the spin galvanic effect indeed exists in Py/AlO$_x$/STO.

Supplementary Note 3: Spin pumping voltage in Py/AlO$_x$/STO multilayers

Figs. S2a and S2b show the optical images of the device and the sample holder. Previously, we have demonstrated that this device configuration is ideal for the separation of the dc voltage induced by the anisotropic magneto-resistance effect V_{AMR} and the dc voltage induced by spin pumping V_{SP} in NM/FM bilayers if an insulating substrate (e.g., Si[22] and GaAs[23]) with a low relative permittivity constant is used. Here, the NM/FM stripe experiences a homogeneous out-of-plane Oersted field excitation h_o generated by the microwave current flowing in the signal and ground lines, and therefore we expect a symmetric $V_{AMR} \sim \sin 2\varphi$, while $V_{SP} \sim \sin\varphi$, where φ is the angle of the magnetization as defined in Fig. 2b. This means that, when $\varphi = \pm 90^{\circ}$ and **H** (**M**) is perpendicular to the stripe, V_{SP} is maximized while $V_{AMR} = 0$, and therefore one can determine V_{SP} accurately. However, this method doesn't apply when using STO as a substrate. This is because STO has a large permittivity (~20000 at 10 K), and a significant shunting microwave current $\mathbf{j}_{STO}$ can be induced in the substrate due to capacitive/inductive coupling[24]. This generates an additional in-plane excitation h_{In} acting on Py. Here, we assume that $\mathbf{j}_{STO}$ forms an angle Φ_{STO} with respect to the stripe direction. The microwave current in Py, $\mathbf{j}_{Py}$, induced by both microwave currents flowing in the CPW and in STO, forms an angle Φ_{Py} with respect to the stripe. Therefore, the symmetric and antisymmetric component of V_{AMR}, V_{AMR}^{S} and V_{AMR}^{A}, are given by

$$V_{AMR}^{S} = V_{S,AMR}^{\parallel}\cos(\varphi - \Phi_{STO})\sin 2(\varphi - \Phi_{Py}) + V_{S,AMR}^{\perp}\sin 2(\varphi - \Phi_{Py}) \qquad \text{(S1)}$$

$$V_{AMR}^{A} = V_{A,AMR}^{\parallel}\cos(\varphi - \Phi_{STO})\sin 2(\varphi - \Phi_{Py}) + V_{A,AMR}^{\perp}\sin 2(\varphi - \Phi_{Py}). \qquad \text{(S2)}$$

Here $V_{S,AMR}^{\parallel}(V_{A,AMR}^{\parallel})$ is the magnitude of the symmetric (anti-symmetric) AMR voltage induced by in-plane excitation, and $V_{S,AMR}^{\perp}(V_{A,AMR}^{\perp})$ the magnitude of the symmetric (anti-symmetric) AMR voltage induced by out-of-plane excitation. Equations S1 and S2 show that: for in-plane excitation, both V_{AMR}^{S} and V_{AMR}^{A} have opposite signs when rotating **M** by 180° (i.e., $\varphi \rightarrow \varphi + 180^{\circ}$), and we obtain

$$\begin{cases} V_{S,AMR}^{\parallel}(+H) = -V_{S,AMR}^{\parallel}(-H) \\ V_{A,AMR}^{\parallel}(+H) = -V_{A,AMR}^{\parallel}(-H) \end{cases}. \tag{S3}$$

While for out-of-plane excitation, both V_{AMR}^{S} and V_{AMR}^{A} have the same sign when rotating **M** by 180°, and we obtain

$$\begin{cases} V_{S,AMR}^{\perp}(+H) = V_{S,AMR}^{\perp}(-H) \\ V_{A,AMR}^{\perp}(+H) = V_{A,AMR}^{\perp}(-H) \end{cases} \tag{S4}$$

On the other hand, the dc voltage induced by spin pumping V_{SP}^{S} is Φ_{Py}-independent, and has a symmetric line shape. For isotropic spin-charge conversion (e.g., by the conventional spin-Rashba effect and the isotropic spin Hall effect), V_{SP}^{S} is proportional to the cross product of the direction of the spin current $\mathbf{J}_S$ (// **z**) and the spin polarization vector $\boldsymbol{\sigma}$ (// **M**), i.e., $V_{SP}^{S} \sim \mathbf{z} \times \boldsymbol{\sigma}$ holds. The φ-dependence of V_{SP}^{S} induced by h_O and h_{In} is given by

$$V_{SP}^{S} = V_{h_{In}} \cos^2(\varphi - \Phi_{STO}) \sin\varphi + V_{h_O} \sin\varphi. \tag{S5}$$

Obviously, V_{SP}^{S} has an odd symmetry with respect to H for both in-plane and out-of-plane excitations, i.e.,

$$V_{SP}^{S}(+H) = -V_{SP}^{S}(-H). \tag{S6}$$

The symmetries of the V_{AMR}^{A}, V_{AMR}^{S} and V_{SP}^{S} spectra with respect to H for both in-plane and out-of-plane excitations are shown in Figure S3. It is noted that it is impossible to disentangle V_{SP}^{S} from V_{AMR}^{S} if the in-plane excitation dominates.

Based on the symmetries of the dc voltages (equations S3, S4 and S6), we have mesured the dc voltage spectra at each magnetization angle φ both along +**H** and –**H**. The magnitudes of $V_{sym}(+H)$, $V_{sym}(-H)$, $V_{a\text{-}sym}(+H)$ and $V_{a\text{-}sym}(-H)$ are obtained. As an example, in Figures S4a and b, we show φ_H-dependence of $V_{sym}(+H)$, $V_{sym}(-H)$ and $V_{a\text{-}sym}(+H)$, $V_{a\text{-}sym}(-H)$ at V_G = +110 V. The magnitude of Φ_{Py}, Φ_{STO} and V_{SP} can be quantified in the following three steps:

I) Since $V_{a\text{-}sym}$ originates only from AMR, based on equations S3 and S4, V_{AMR}^{A} induced by h_O can be determined by

$$V_{AMR}^{A,h_O} = \frac{V_{a-sym}(-H) - V_{a-sym}(+H)}{2} \propto V_{A,AMR}^{\perp} \sin 2(\varphi - \Phi_{Py}), \tag{S7}$$

In this case, any $V_{a\text{-}sym}$ induced by h_I can be excluded. As shown in Figure S5a, the angular trace can be well fitted by equation S7 and Φ_{Py} of –5° is obtained.

II) Similarly, V_{AMR}^{A} induced by h_I can be determined by adding $V_{a\text{-}sym}(-H)$ and $V_{a\text{-}sym}(+H)$, i.e.,

$$V_{AMR}^{A,h_I} = \frac{V_{a-sym}(-H) + V_{a-sym}(+H)}{2} \propto V_{A,AMR}^{\parallel} \cos(\Phi_{STO} - \varphi) \sin 2(\varphi - \Phi_{Py}) \tag{S8}$$

As shown in Figure S5b, by using the corresponding Φ_{Py} value (−5°) obtained in Fig. S5a, the φ-dependence of V_{AMR}^{A,h_I} can be well fitted by equation S8, and Φ_{STO} is determined to be −60°.

III) The spin pumping voltage V_{SP}^{S} can be determined by substracting $V_{\text{sym}}(-H)$ and $V_{\text{sym}}(+H)$

$$V_{SP}^{S} = \frac{V_{sym}(-H) - V_{sym}(+H)}{2} \qquad \text{(S9)}$$

In this case, symmetric voltages originating from AMR induced by h_O can be excluded. As shown in Figure S5c, the angular trace of V_{SP}^{S} shows a sinφ dependence, which confirms the existence of a spin pumping voltage. However, for other gate-voltages, the angular traces cannot be simply fitted by sinφ (not shown). There could be two reasons for this: I) the in-plane driving dominates, and it is impossible to separate the spin pumping voltage from the symmetric AMR effect, and II) an anisotropic spin and/orbital distribution of the AlO_x/STO 2DEG[20,21]. At present, we don't know how to separate the orbital contribution from the spin contribution, and we will leave this for further studies.

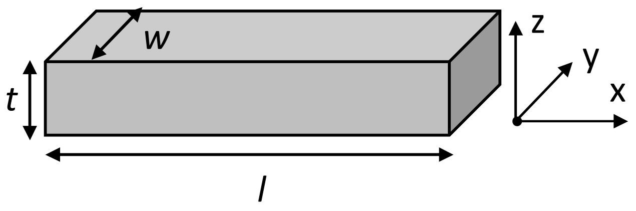

Fig. S6. Rectangular magnetic stripe with length *l,* width *w,* and thickness *t* used to calculate the shape anisotropy.

Supplementary Note 4: Quantifying the shape anisotropy of the Py/AlO$_x$/STO stripe

Since the size of our device is l = 320 um, w = 7 um and t = 6 nm, it can be treated as an infinitely long stripe. The demagnetization tensor **N** is given by[S1]

$$\mathbf{N} = \begin{pmatrix} 0 & 0 & 0 \\ 0 & N_{yy} & 0 \\ 0 & 0 & N_{zz} \end{pmatrix} \qquad \text{(S10)}$$

where N_{yy} and N_{zz} are, respectively, the demagnetization factors along the *y*- and *z*-directions as defined in Fig. S6, and $N_{yy} + N_{zz} = 1$ holds. N_{yy} only depends on the aspect ratio $p = w/t$, and is given by

$$N_{yy}(p) = \frac{1}{\pi}\left[\frac{1-p^2}{2p}\ln(1+p^2) + p\ln p + 2\tan^{-1}\frac{1}{p}\right] \quad \text{(S11)}$$

Therefore, N_{yy} is determined to be 0.0023 for p = 1167, and the uniaxial anisotropic field is $\mu_0 H_U = \mu_0 N_{yy} M_S$ = 2.1 mT when using $\mu_0 M_S$ = 0.92 T as determined by magnetization measurements. And the magnitude of $\mu_0 H_K = \mu_0 N_{zz} M_S$ = 914 mT. Both $\mu_0 H_K$ and $\mu_0 H_U$ quantitatively match the value determined by FMR measurements (Figs. 3a and 3b).

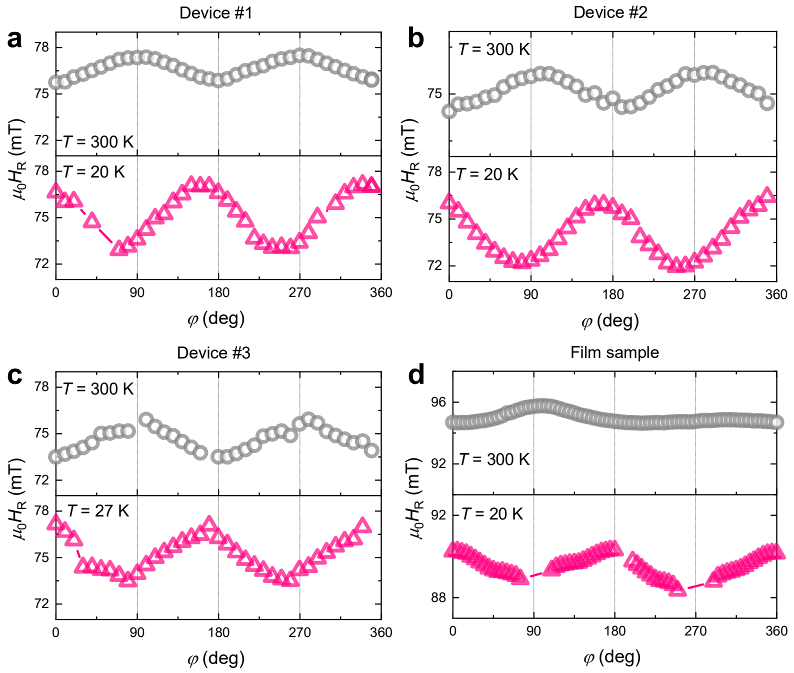

Fig. S7. (**a**), (**b**) and (**c**): φ-dependence of H_R for other Py/AlO$_x$/STO stripe devices measured at f = 8 GHz. (**d**) φ-dependence of H_R measured for unpatterned Py/AlO$_x$/STO thin film.

Supplementary Note 5: Angular dependence of H_R for other Py/AlO$_x$/STO samples

To establish the replicability for the phase shift of the H_R-trace, another two stripe devices (Device 2 and 3) have been measured. As shown in Figs. S7a-S7c, similar phases shift has been observed, indicating the phase shift is a genuine effect. Moreover, we have also measured unpatterned Py/AlO$_x$/STO thin film. As shown in Fig. S7d, the H_R-trace is almost constant for T = 300 K, and this is expected that unpatterned sample has no in-plane uniaxial anisotropy. However, as T

decreases to 20 K, a shift of H_R is observed, being consistent with the stripe device. Therefore, we conclude that the H_R-shift at low temperature is universal, independent of the shape of the measured device.

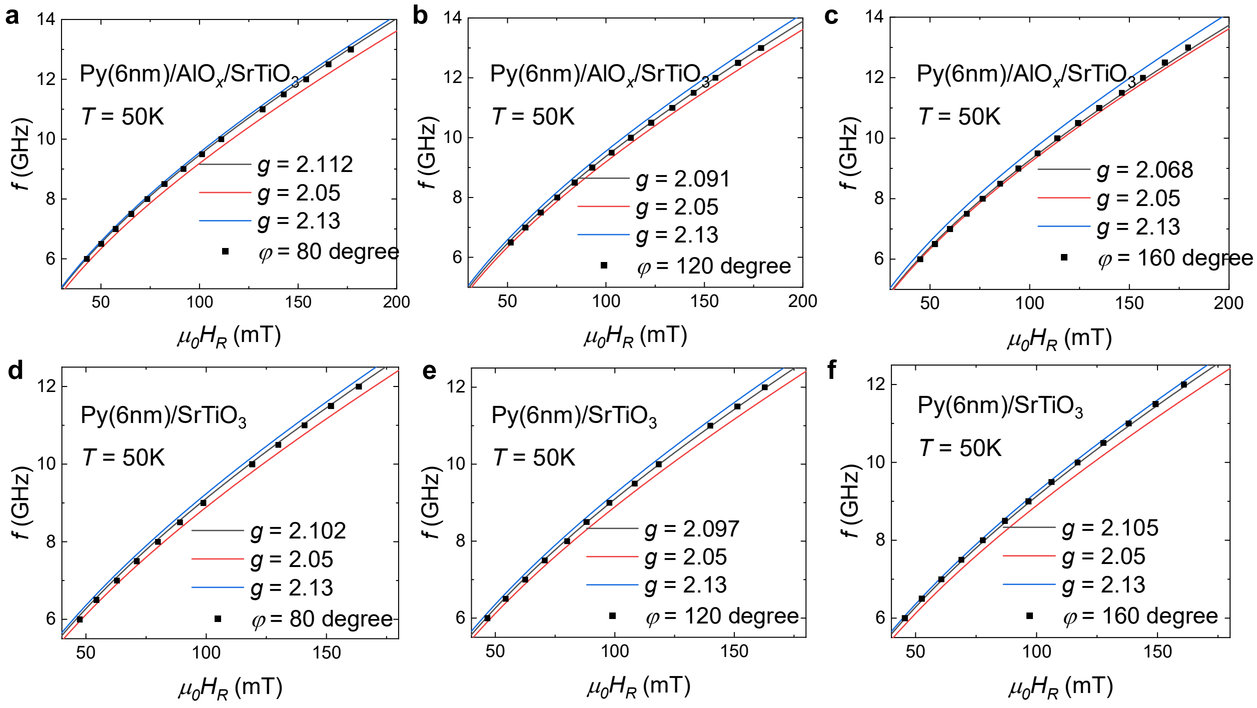

Fig. S8. (**a**), (**b**) and (**c**): f-dependence of H_R for the Py/AlO$_x$/STO device measured at $\varphi = 80^o$, $\varphi = 120^o$ and $\varphi = 160^o$. (**d**), (**e**) and (**f**): f-dependence of H_R for the Py/STO device measured at $\varphi = 80^o$, $\varphi = 120^o$ and $\varphi = 160^o$. The solid lines are calculated by using the corresponding g-values as indicated. For the Py/AlO$_x$/STO device, anisotropic g-values are needed to explain the experimental results; while for the Py/STO device, only an isotropic g-value is needed.

Supplementary Note 6: Frequency dependence of H_R in Py/AlO$_x$/STO and Py/STO devices

Fig. S8a, S8b and S8c show the f-dependence of H_R for the Py/AlO$_x$/STO device measured at selective angles of $\varphi = 80^o$, $\varphi = 120^o$ and $\varphi = 160^o$. To fit the experimental data, anisotropic g-values are needed; however, for the Py/STO device, an isotropic g-value about 2.1 is obtained for all the measured angles.

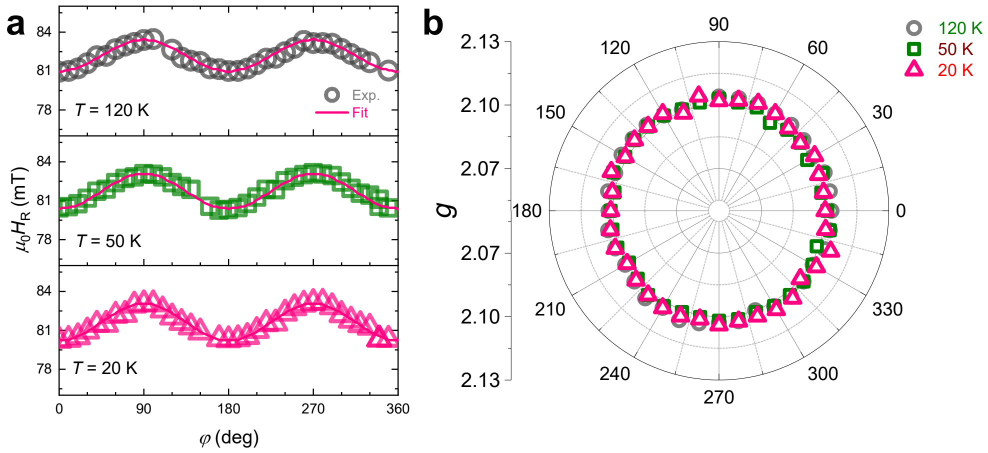

Fig. S9. (**a**) φ-dependence of H_R for the Py/Pt/STO sample measured at $f = 8$ GHz and at $T = 120$ K, 50 K, and 20 K. The solid lines are fits by equation 4. (**b**) Polar plot of the isotropic g-factor at different temperatures.

Supplementary Note 7: Isotropic *g*-factor in Py/Pt/STO

Figure S9a shows the φ-dependence of the resonance field H_R measured at $f = 8$ GHz and $T = 120$ K, 50 K, and 20 K for the Py/Pt/STO sample. The H_R-trace shows typical uniaxial anisotropies due to the shape anisotropy, and an isotropic g-factor is obtained at all measured temperatures. These results indicate that Py (Pt) is an isotropic spin source (sink).

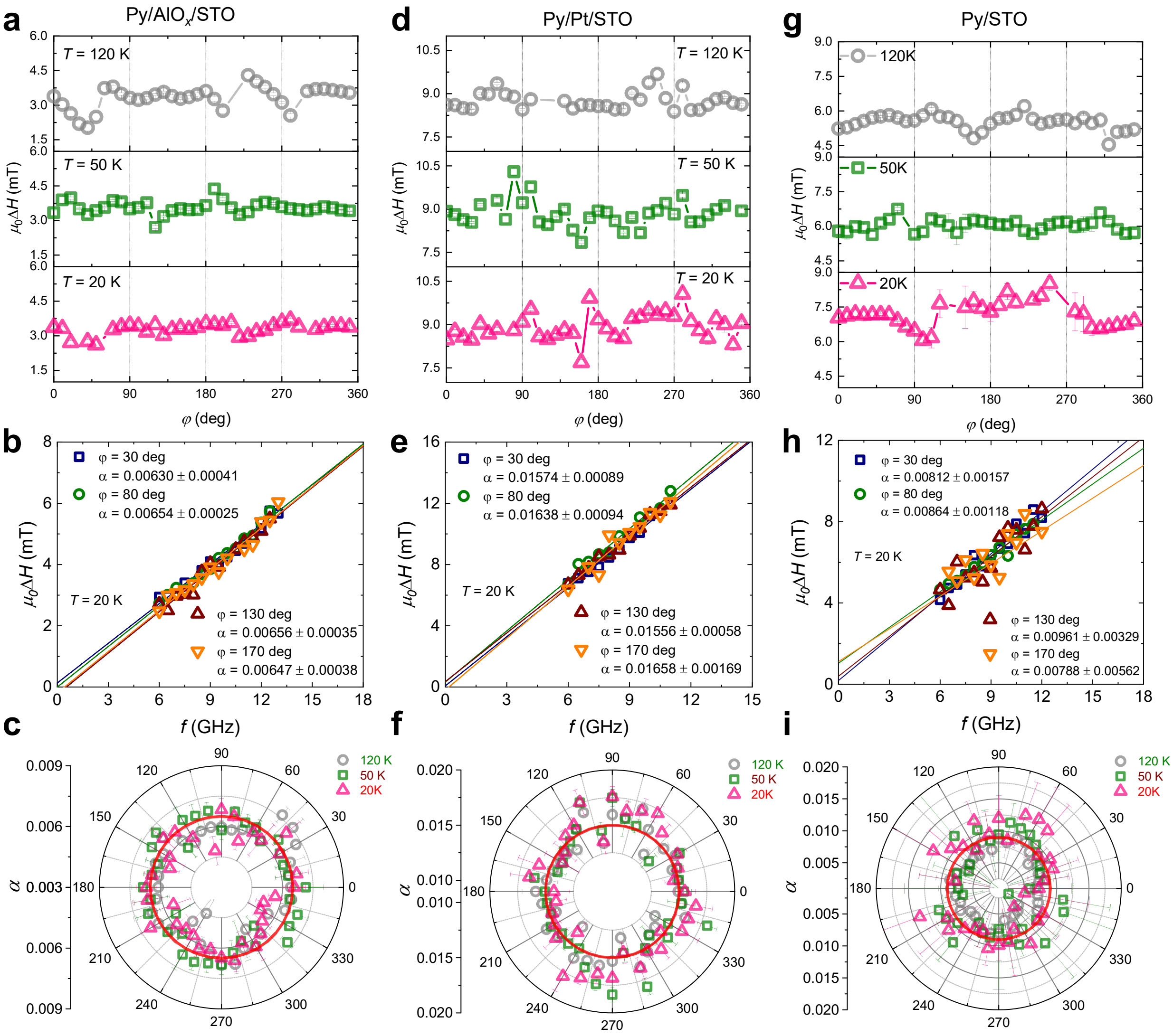

Fig. S10. (**a**) φ-dependence of the linewidth ΔH of a Py/AlO$_x$/STO sample measured at f = 8 GHz and at T = 120 K, 50 K, and 20 K. (**b**) f-dependence of ΔH of a Py/AlO$_x$/STO sample for φ = 30°, 80°, 130° and 170°. (**c**) Polar plot of the damping values for Py/AlO$_x$/STO samples at different temperatures. (**d**), (**e**) and (**f**): The same plots as (**a**), (**b**) and (**c**) but for Py/Pt/STO sample. (**g**), (**h**) and (**i**): The same plots as (**a**), (**b**) and (**c**) but for Py/STO sample.

Supplementary Note 8: Angular- and temperature-dependence of damping in Py/AlO$_x$/STO, Py/STO and Py/Pt/STO

Figure S10a shows the φ-dependence of the linewidth ΔH measured at f = 8 GHz and T = 120 K, 50 K, and 20 K

for the Py/AlO$_x$/STO sample. The magnitude of ΔH shows a weak variation at low temperatures. The f-dependence of ΔH for selected $\varphi = 30^{\circ}$, 80°, 130°, and 170° is shown in Fig. S10b. The magnitude of the damping value α at each φ can be quantified by

$$\mu_0 \Delta H = 2\alpha \frac{2\pi f}{\gamma} + \mu_0 \Delta H_0 \tag{S12}$$

Here ΔH_0 is the zero-frequency offset of the linewidth. By using the anisotropic g-factors obtained in Fig. 3c, the corresponding α values at each φ are determined. These results show that, within the experimental accuracy, the damping is almost isotropic (Fig. S10c) for Py/AlO$_x$/STO. Similarly, the same measurements are performed for Py/Pt/STO and Py/STO samples. As shown in Figs. S10 d-i, an isotropic damping is obtained. Moreover, for all the measured samples, no significant enhancement of damping is observed at low temperature.

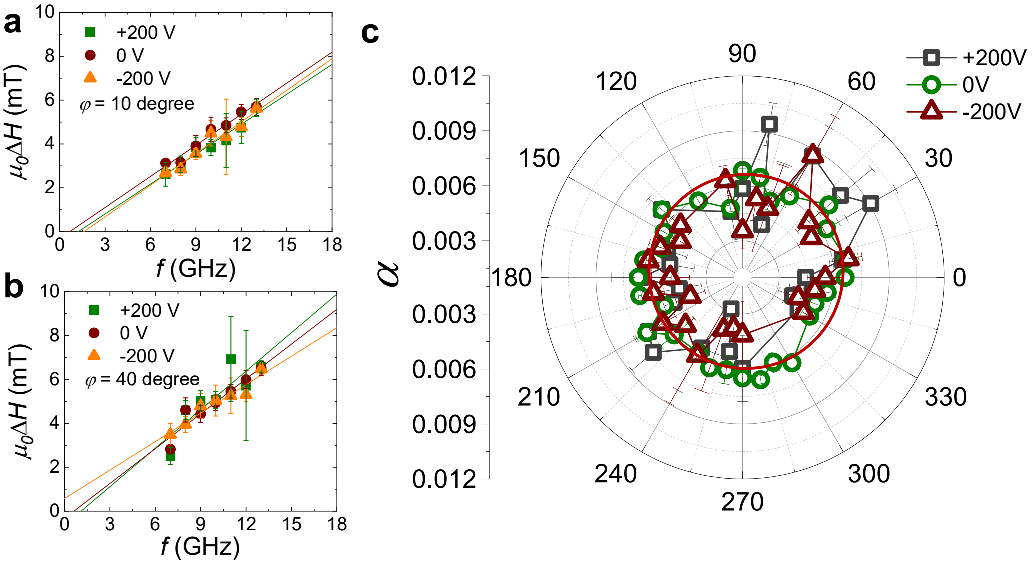

Fig. S11. (**a**) f-dependence of ΔH for various gate voltages for Py/AlO$_x$/STO measured at $\varphi = 10^{\circ}$. (**b**) f-dependence of ΔH for various gate voltages for Py/AlO$_x$/STO for $\varphi = 40^{\circ}$. (**c**) Polar plot of damping which shows no clear modification of damping by back-gate voltages.

Supplementary Note 9: V_G-dependence of damping in Py/AlO$_x$/STO

Figures S11a and S11b respectively present the f-dependence of ΔH measured at V_G = +200 V, 0 V and -200 V for $\varphi = 10^{\circ}$ and 40°. Within the experimental error bar, the damping does not change with V_G. The angular dependence of

damping for V_G = +200 V, 0 V, and –200 V is shown Figure S11c, and no clear modulation of damping by V_G is observed.

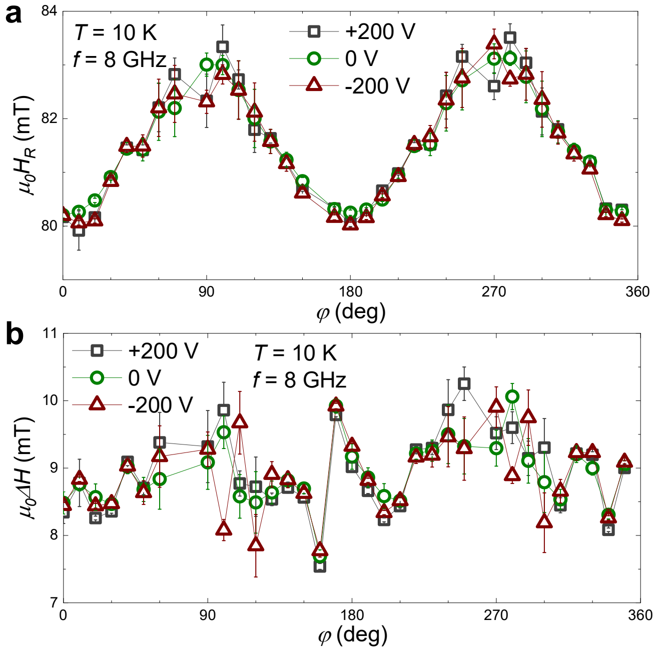

Fig. S12. (**a**) φ-dependence of H_R for the Py/Pt/STO sample V_G = +200 V, 0 V, and –200 V. (**b**) φ-dependence of ΔH for the Py/Pt/STO sample V_G = +200 V, 0 V, and –200 V. There is no modulation for H_R and ΔH.

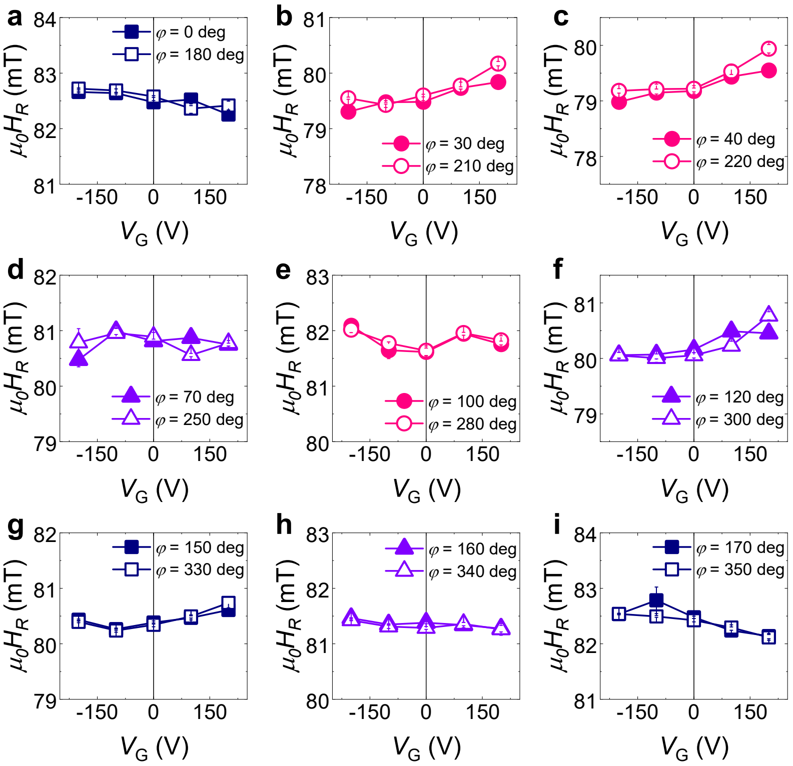

Fig. S13. V_G-dependence of H_R for the Py/STO sample measured at different angles. A weak anisotropic modulation is observed. For example, a positive slope ~ +0.25 mT/100 V is observed for φ = 40° and 220°; a negative slope ~ −0.2 mT/100 V is observed for φ = 0° and 180°; φ = 160° and 340°, H_R is independent on V_G.

Supplementary Note 10: V_G-dependence of resonance field and damping in Py/Pt/STO and Py/STO

The effects of the electric-field on H_R and ΔH for the Py/Pt/STO sample are, respectively, shown in Fig. S12a and b. The application of gate-voltage does not change H_R and ΔH of the Py/Pt/STO sample, where the spin- and orbit-currents are blocked by Pt. While for Py/AlO_x/STO where the spin- and orbit-currents are not blocked, sizeable modification of H_R is observed. This confirms the importance of the 2DEG for the modification of the anisotropic g-factor. While for the Py/STO sample as shown in Fig. S13, a much weaker modulation is observed because of a much weaker 2DEG at the Py/2DEG interface[26].

Supplementary Note 11: Excluding the charge mediated magnetoelectric coupling effect in gate modulation of measurement

A previous work[28] has measured the voltage induced H_R shift in NiFe (0.6 nm – 1.2 nm)/$SrTiO_3$ (50 nm)/Pt capacitor structures. Here by applying an electric-field of ~1 MV/cm, a maximal H_R shift of 6.5 mT is realized, and the modulation is induced by charge mediated magnetoelectric coupling. However, this mechanism seems unlike for our device. This is because our Py (6 nm) is much thicker and the applied electric field is much weaker (~10^5 V/m), and in this case the magnetic anisotropy of Py can be hardly modulated. Moreover, the magnetoelectric coupling gives an isotropic modulation of HR while our modulation by V_G is highly anisotropic. Therefore, we conclude that our modulation in Py/AlO_x/STO 2DEG is due to the modulation of spin and orbital pumping efficiency, which is conceptually different from the magnetoelectric coupling effect.

References

S1. Henry, Y., Gladii, O., & Bailleul, M. Propagating spin-wave normal modes: A dynamic matrix approach using plane-wave demagnetizating tensors. *arXiv:* 1611. 06153v1. Submitted on 18 Nov 2016. https://doi.org/10.48550/arXiv.1611.06153
